# Supplementary material for: Persistent Illegal Hunting of Wildlife in an African Landscape: Insights from a Study in the Luangwa Valley, Zambia
Source: Animals (Basel). 2024 Aug 19;14(16):2401. doi: 10.3390/ani14162401 (PMC11350902; doi:10.3390/ani14162401)
Supplement: Supplementary file 1 [file animals-14-02401-s001.zip › Form S1.pdf]

Form S1. Informed Consent Form for Participants in a Study in the Luangwa Valley.

ID no……

INFORMED CONSENT FORM FOR PARTICIPANTS IN THE STUDY

Dear participant, through this informed consent form I seek your consent to participate in the academic study in Environmental Management by responding to the questionnaire or participating in group discussions or in-depth interviews. Before you append your consent to participate in the study, read through the details of the researcher and study and if you have no objections you may proceed to consent participation by signing at the end of the form.

Identity and Address of the Researcher

Paul Zyambo
PhD Candidate in Environmental Management
University of Lusaka
LUSAKA

Residential Address: Arduini Farm, Plot 31, Chilanga. Mobile Phone: +260978290175

Research Ethics Committee Address: Chairman of UNILUS REC, P.O. Box 36711, Lusaka. Phone: +260211233407

Purpose of the Study

The purpose of the study is to establish an understanding on persistent illegal hunting among local communities in the Luangwa Valley, Zambia.

Confidentiality

- Information provided by any participant will be treated as confidential. Therefore, the participant is not required to indicate their names on the questionnaires and the names of participants will not be used by the researcher.
- Information provided by the participant on the questionnaire and any recording during focus group discussions will be accessible to only the researcher for data analysis and interpretation.
- Data and records from participants will be kept and secured by only the researcher and will only be disposed of by shredding after five years from the date of collection.
- Information provided by participants will not be used to arrest, prosecute or investigate participants.

Benefits and Risks of Participating in the Study

- One of the benefits of participating in the study is that participants will contribute to enlightening the world on the realities of illegal hunting phenomenon in the Luangwa Valley.

- Another benefit is that participants will contribute to providing information for solving illegal hunting problem that is being addressed in the study.

The risk posed by the study is that participants may suffer fear of being arrested, prosecuted, penalised or disciplined for providing information requested in the questionnaires and focus group discussions.

(As indicated earlier, confidentiality is guaranteed to participants and no information will be used against them to arrest, prosecute, penalise or discipline them).

Rights of Participants

Participants in the study reserve the right to refuse providing any information, avoid responding to some questions and withdraw from participating in the study whenever they feel necessary.

Consent to Participate

Having read and understood the contents of this ***Informed Consent Form for Participants in the Study,*** I agree to participate in the study and will provide honest information to the best of my knowledge and experience.

Signed ………………………………………… Date …………………………………………

Thank You Very Much
