# Supplementary material for: Persistent Illegal Hunting of Wildlife in an African Landscape: Insights from a Study in the Luangwa Valley, Zambia
Source: Animals (Basel). 2024 Aug 19;14(16):2401. doi: 10.3390/ani14162401 (PMC11350902; doi:10.3390/ani14162401)
Supplement: Supplementary file 1 [file animals-14-02401-s001.zip › Guide S1.pdf]

Guide S1. Guide for focus group discussions for a study in the Luangwa Valley.

GUIDE FOR FOCUS GROUP DISCUSSIONS

This serves as a guide for focus group discussions in the study that is aimed at establishing an understanding on persistent poaching among local communities in the Luangwa Valley.

---

1. Participants in Focus Group Discussion

Each Focus Group Discussion (FGD) will comprise about 10 participants who are peers and have something in common that is connected to the study topic or objective. Participants in the FGD will have agreed to and signed the Informed Consent Form to participate in the study. Each participant should be given an opportunity to contribute their views and experiences.

The FGD will be facilitated by a moderator of facilitator. A recorder will record the proceedings of the FGD.

2. Illegal Hunting (Poaching) Levels, Trends, Persistence & Drivers (Motivators) – 10 minutes

The FGD will begin by discussing the levels, trends and persistence of illegal hunting and what drives or motivates local people to engage in illegal hunting in the area.

3. Attitude, Subjective Norms & Perceived Behavioural Control on Illegal Hunting Behaviour – 10 minutes

The FGD will discuss whether poaching is acceptable in some situations (behavioural beliefs), whether immediate families and communities would accept poaching in some situations (normative beliefs) and whether individuals themselves have power to poach or not to (control beliefs).

4. Intervention Measures being Used to Address Drivers of Illegal Hunting – 10 minutes

The FGD will discuss the current intervention measures being employed to address drivers or motivators of illegal hunting and how these measures were performing in addressing the drivers of illegal hunting in the area.

5. Preferences of Intervention Measures to Address Drivers of Illegal Hunting – 10 minutes

The FGD will discuss the preferred intervention measures to address drivers or motivators of illegal hunting in the area.

6. Discuss and Clarify Relevant Issues on Illegal Hunting - 10 minutes

The FGD will discuss and clarify any other important aspects of illegal hunting, drivers of illegal hunting, intervention measures and performance that may have arisen or emerged from the quantitative method of the study.

Thank You Very Much
