# Supplementary material for: Persistent Illegal Hunting of Wildlife in an African Landscape: Insights from a Study in the Luangwa Valley, Zambia
Source: Animals (Basel). 2024 Aug 19;14(16):2401. doi: 10.3390/ani14162401 (PMC11350902; doi:10.3390/ani14162401)
Supplement: Supplementary file 1 [file animals-14-02401-s001.zip › Guide S2.pdf]

Guide S2. Guide for In-depth interviews for the study in the Luangwa Valley

GUIDE FOR IN-DEPTH INTERVIEWS

This serves as a guide for in-depth interviews in the study that is aimed at establishing an understanding on persistent illegal hunting among local communities in the Luangwa Valley.

A. Make the interviewee comfortable by introducing the study and indicate why they have been included for an in-depth interview

The interviewees will be individuals who are experienced in matters relating to poaching, intervention measures and conservation of wildlife in the Luangwa Valley for at least 10 years and are able to share their views and experiences.

Participants will have agreed to and signed the Informed Consent Form to participate in the study and to be recorded.

The researcher will conduct in-depth interviews and record the proceedings accordingly.

B. Guideline questions for the in-depth interviews

The following will be the main questions for in-depth interviews, with start-up and follow-up questions being asked for background, further details and clarification:

1. In your view what is the status (levels, trends and persistence) of poaching among local communities in the Luangwa Valley and what is driving this poaching?

2. Why has poaching persisted among local communities in the Luangwa Valley despite intervention measures such as law enforcement, community conservation approach, conservation education, etc.?

3. Do you think the current intervention measures are adequate for addressing poaching among local communities in the Luangwa Valley?

4. What should be done to eliminate or control poaching among local communities in the Luangwa Valley?

Thank You Very Much
