# Supplementary material for: Persistent Illegal Hunting of Wildlife in an African Landscape: Insights from a Study in the Luangwa Valley, Zambia
Source: Animals (Basel). 2024 Aug 19;14(16):2401. doi: 10.3390/ani14162401 (PMC11350902; doi:10.3390/ani14162401)
Supplement: Supplementary file 1 [file animals-14-02401-s001.zip › Letter S1 .pdf]

Letter S1. Approval letter by the Research Ethics Committee of the University of Lusaka.

UNILUS

UNIVERSITY of LUSAKA

Plot No. 37413, Off Alick Nkhata Mass Media, P. O. Box 36711, Lusaka
Phone: +260 211 233407, 258409, Fax: +260 211 233409, E-mail: ictar@zamnet.zm, unilus@zamnet.zm
Website: www.unilus.ac.zm

All correspondence should be addressed to the Vice Chancellor

14th October 2020

Mr. Paul Zyambo

Arduini Farm, Plot no. 31

Chilanga, Lusaka

Zambia

RESPONSE LETTER

IORG0010092-0308/20: *Analysis of Underlying Drivers of Illegal Hunting and MitigationMeasures in the Luangwa Valley, Zambia*

Dear Mr. Paul Zyambo:

This letter serves to inform you that the UNILUS REC reviewed your research proposal submitted in September 2020.

The proposal was approved subject to minor corrections.

The minor issues identified are itemized in the report attached.

You are required to address the issues raised by the reviewer. The regulations of UNILUS REC require that these corrections be done within 2 weeks. You will be expected to write a letter listing the corrections that would have been made in the proposal. This should be submitted to the secretary of Research Ethics Committee for our records.

Wishing you well in your research.

Signature...

Professor Kasonde Bowa

Chairman of UNILUS REC.

UNILUS
