# Supplementary material for: Persistent Illegal Hunting of Wildlife in an African Landscape: Insights from a Study in the Luangwa Valley, Zambia
Source: Animals (Basel). 2024 Aug 19;14(16):2401. doi: 10.3390/ani14162401 (PMC11350902; doi:10.3390/ani14162401)
Supplement: Supplementary file 1 [file animals-14-02401-s001.zip › Questionnaire S2.pdf]

Questionnaire S2. Questionnaire for Wildlife Agency Staff/Community Resource Boards/Conservation Interested Entities in the Luangwa Valley.

ID no……

QUESTIONNAIRE FOR WILDLIFE AGENCY STAFF/ COMMUNITY RESOURCE BOARDS/ CONSERVATION INTERESTED ENTITIES IN THE LUANGWA VALLEY

Dear participant, this questionnaire survey is part of the university study in environmental management and please be assured that your personal responses will be considered confidential and will not be used against you whatsoever. You are further assured that you reserve the right to respond and withhold any response whenever you feel so. Please do not tell any of your colleagues your responses in this questionnaire.

Date: .... Area/Game Management Area: ………..

Personal Data

1. What is your gender/sex?

Female ….	Male ….

2. What is your age group? Choose (tick) **one** correct response.

   i. Less than 30 years old……
   ii. 30 – 39 years old........
   iii. 40 – 50 years old…….
   iv. More than 50 years old………

3. Would you consider yourself as:

   i. Very wealthy/rich …….?
   ii. Moderately wealthy/rich …….?
   iii. Poor ……….?
   iv. I don’t know ……

4. Do you have any source of income?

Yes …...	No ……

5. If your response was **Yes** in question (4) above, indicate the main source of income.

   i. Employment. ………..
   ii. Farming. ………….
   iii. Tourism activities. …………..
   iv. Hunting activities. ………..
   v. Business activities. ……….
   vi. Any other source (please indicate). ....

6. From the sources of your income indicated in question 4 above, indicate your income levels.

   i. Less than K1,000 per month ……….
   ii. K1,000 – 4,900 per month ……….
   iii. K5,000 – K10,000 per month ……….
   iv. More than K10,000 per month ………

7. What is highest educational level that you have attained? Choose (tick) **one** correct response.

    i. None ……….
    ii. Primary school level ………
    iii. Junior secondary school level ………
    iv. Senior secondary school level ………
    v. Tertiary (college/university) level ……..

Levels, Trends and Persistence of Poaching Activities

8. Basing on your knowledge and experience, how would you describe the level of poaching activities in this area? Choose (tick) **one** correct response.

Low…….	Moderate…….	High……….

9. What could be the main reason for your response in question 8 above?

   If your response is **Low** in question 8 above, choose or tick **one** correct reason:

    i. Consumption of illegal bushmeat is **not** common in this area. ………..
    ii. Use of firearms/wire snares to kill animals is **not** common in this area. ………..
    iii. Selling of illegal bushmeat is **not** common in this area. ……….
    iv. Observed incidences of local people arrested for poaching are **not common**. ……
    v. Observed animal populations are **very** abundant in the area. …………

   If your response is **Moderate** in question 8 above, choose or tick **one** correct reason:

    i. Consumption of illegal bushmeat is **moderately** common in this area. ………..
    ii. Use of firearms or wire snares to kill animals is **moderately** common in this area. …..
    iii. Selling of illegal bushmeat is **moderately** common in this area. ……..
    iv. Observed incidences of local people arrested for poaching are **moderately** common. ……..
    v. Observed animal populations are **moderately** abundant in the area. ……..

   If your response is **High** in question 8 above, choose or tick **one** correct reason:

    i. Consumption of illegal bushmeat is **very** common in this area. ………..
    ii. Use of firearms/wire snares to kill animals is **very** common in this area. ………..
    iii. Selling of illegal bushmeat is **very** common in this area. ……….
    iv. Observed incidences of local people arrested for poaching are **very** common. ………
    v. Observed animal populations are **not** abundant in the area. …………

10. Basing on your knowledge and experiences, how long has the poaching level you indicated in **question 8** been going on? Choose (tick) **one** correct response.

    i. Poaching has just started in the area. …….
    ii. Short time (not more than 15 years) ……..
    iii. Long time (from 15 to 30 years) ……..
    iv. Very long time (over 30 years) ………

11. What could be the main reason for the poaching level to continue for the period you chose in question 10 above? Choose (tick) **one** correct response.

i. Motivating or driving factors for poaching have been there during the said period …….
ii. There were **No** motivating or driving factors for poaching during the said period ……..
iii. Effective intervention measures against poaching were there during the said period ……..
iv. There were **No** effective intervention measures against poaching during the said period ……..

12. Basing on your knowledge and experience, how would you describe the general trends of poaching activities in this area? Choose (tick) **one** correct response.

Decreased……	Stable……	Increased……

13. What could be the main reason for your response in question 12 above?

If your response is **Decreased** in question 12 above, choose or tick **one** correct reason:

i. Consumption of illegal bushmeat has **reduced** in this area. ………..
ii. Use of firearms/wire snares to kill animals has **reduced** in this area. ………..
iii. Selling of illegal bushmeat is has **reduced** in this area. ……….
iv. Observed incidences of local people arrested for poaching have **reduced**. ……..
v. Observed animal populations have **increased** in abundance in the area. …………

If your response is **Stable** in question 12 above, choose or tick **one** correct reason:

i. Consumption of illegal bushmeat has **remained stable** in this area. ………..
ii. Use of firearms or wire snares to kill animals has **remained stable** in this area. …
iii. Selling of illegal bushmeat has **remained stable** in this area. ……….
iv. Observed incidences of local people arrested for poaching have **remained stable** …......
v. Observed animal populations have **remained stable** in abundance in this area. ….

If your response is **Increased** in question 12 above, choose or tick **one** correct reason:

i. Consumption of illegal bushmeat has **increased** in this area. ………..
ii. Use of firearms/wire snares to kill animals has **increased** in this area. ………..
iii. Selling of illegal bushmeat has **increased** in this area. ……….
iv. Observed incidences of local people arrested for poaching have **increased**. ………
v. Observed animal populations have **reduced** in abundance in the area. …………

Drivers/Motivations for Poaching

14. What drives/motivates local people to poach wildlife in this area? First look at the list below and then choose (tick) **ANY as your most important correct responses**:

i. Preventative killing (preventing animals from killing someone or damaging property). ....
ii. Retaliatory killing (killing animals because they damaged crops or property or killed someone) …….
iii. Human-wildlife conflicts ………
iv. Traditional and cultural needs (for spirits, ancestors, ceremonies) ………
v. Need for trophies (horns, skins, bones and medicine (non-meat) ……..
vi. Lack of tangible benefits from wildlife conservation ……..
vii. Lack of alternative sources of income/employment ……...
viii. Lack of alternative sources of meat/protein. ………
ix. Need for income through trade in bushmeat and wildlife parts. ……...
x. Inadequate/weak law enforcement (patrols) and regulation. …………
xi. Need for bushmeat for consumption/food security. ……….
xii. Poverty (lacking basic sources of sustenance and livelihood). …….
xiii. Protesting against unfair or unjust rules/regulations on wildlife…….
xiv. High market demand for bushmeat/ivory/skins. ……..
xv. Lack of education/awareness of consequences of poaching to animals and poachers. ….
xvi. Inadequate involvement of communities in wildlife management with lack of rights over wildlife. ……
xvii. Political influence. …….
xviii. Joy or thrill of killing animals. ……..
xix. Recreational (relaxation) needs…….
xx. Rich outsiders sponsor local people to poach. ………
xxi. Desire to outsmart law enforcement…….
xxii. Firearm (shooting) training/practice…….
xxiii. Human population increase/influx. ……..
xxiv. If not in the list, please indicate:
…………………………………………………………………………………………………
……………………………………………………………………………………………

Intervention Measures

15. What intervention measures **are being implemented** to address what drives/motivates poachers to go poaching in this area (in reference to your responses in question 14 above)? First look at the list below and then choose (tick) **ANY as your important correct responses**:

    i. Provision of conservation awareness/education/sensitisation programmes. ……
    ii. Provision of alternatives to bushmeat ……..
    iii. Anti-poaching law enforcement efforts……..
    iv. Revenue sharing from trophy hunting………
    v. Bushmeat provision through hunting……….
    vi. Involving communities in wildlife management. ……..
    vii. Provision of alternative livelihoods………
    viii. Provision of alternative sources of employment/income. …….
    ix. Protection of communities from wildlife threats and attacks……..
    x. Provision of access to needed wildlife resources. ………
    xi. Land use planning. ……..
    xii. If not on the list, please indicate what is being implemented
    ……………………………………………………………………………………………
    ……………………………………………………………………………………………

16. If you **were** a project manager in this area, how would you assess the impact of the intervention measures **you identified above (question 15)** on drivers/motivators of poaching? Choose (tick) **one** correct response for **each** identified measure.

i. Provision of conservation awareness/education/sensitisation programmes.
Unsatisfactory……. Fairly satisfactory…….. Most satisfactory………

Reason for your assessment. Choose (tick) **one** correct reason for the assessment.
Poorly/not widely done………. Fairly/widely done……… Well/widely done ………

ii. Provision of alternatives to bushmeat and poaching.
Unsatisfactory……. Fairly satisfactory…….. Most satisfactory………

Reason for your assessment. Choose (tick) **one** correct reason for the assessment.
Poorly/not done……. Fairly/widely done …….. Well /widely done ………

iii. Anti-poaching law enforcement efforts.
Unsatisfactory……. Fairly satisfactory…….. Most satisfactory………

Reason for your assessment. Choose (tick) **one** correct reason for the assessment.
Inadequate resources/legislation…… Fair resources/legislation…….. Adequate resources/legislation………

iv. Revenue sharing from trophy hunting.
Unsatisfactory……….. Fairly satisfactory……… Most satisfactory………..

Reason for your assessment. Choose (tick) **one** correct reason for the assessment.
Improperly/inadequate sharing….. Fairly adequate sharing…. Adequate sharing ….

v. Bushmeat provision from hunting.
Unsatisfactory………. Fairly satisfactory……….. Most satisfactory………….

Reason for your assessment. Choose (tick) **one** correct reason for the assessment.
Improperly/inadequate ……. Fairly adequate provision…… Adequate provision……

vi. Involving communities in wildlife management.
Unsatisfactory………. Fairly satisfactory………. Most satisfactory…………

Reason for your assessment. Choose (tick) **one** correct reason for the assessment.
Limitedly involved………. Fairly involved……….. Adequately involved………….

vii. Provision of alternative livelihoods.
Unsatisfactory……….. Fairly satisfactory………… Most satisfactory………….

Reason for your assessment. Choose (tick) **one** correct response for the assessment.
Limitedly provided……. Fairly provided…….. Adequately provided………

viii. Provision of alternative sources of employment/income.
Unsatisfactory………. Fairly satisfactory………. Most satisfactory………..

Reason for your assessment. Choose (tick) **one** correct reason for the assessment.
Limitedly provided……….. Fairly provided………… Adequately provided………..

ix. Protection of communities from wildlife threats and attacks.
Unsatisfactory………. Fairly satisfactory……….. Most satisfactory………….

Reason for your assessment. Choose (tick) **one** correct reason for the assessment.
Late/poorly done………. Fairly done…….. On time and well done………

x. Provision of access to needed wildlife resources.
Unsatisfactory……. Fairly satisfactory…….. Most satisfactory………

Reason for your assessment. Choose (tick) **one** correct reason for the assessment.
Limitedly/not provided……. Fairly provided…….. Well provided………

xi. Land use planning.
Unsatisfactory……. Fairly satisfactory…….. Most satisfactory……….

Reason for your assessment. Choose (tick) **one** correct reason for the assessment.
Not planned/implemented……. Fairly implemented…….. Well implemented………

xii. For those not on the list, please write and indicate how they are impacting on drivers/motivators of poaching.
……………………………………..…………………………………………………………
……………………………………………………………………………………………………

17. If you **were** a manager of wildlife in the area, what intervention measures would you **prefer** implemented to address the drivers/motivators of poaching among the community members? First look at the list below and then choose (tick) **ANY but NOT more than 5 as your important preferences**:

i. Enhancing conservation awareness/education/sensitisation programmes. ……
ii. Provision of alternatives to bushmeat ……..
iii. Increasing or strengthening anti-poaching law enforcement efforts……
iv. Improving revenue sharing from trophy hunting……….
v. Improving bushmeat provision from hunting………
vi. Introducing benefit sharing of revenues from National Parks…………
vii. Improving community involvement in wildlife management. ……..
viii. Enhancing provision of alternative livelihoods………
ix. Enhancing provision of alternative sources of employment/income. …….
x. Improving protection of communities from wildlife threats and attacks……..
xi. Provision of access to needed wildlife resources. ………
xii. Improving implementation of land use plans. ……..
xiii. Removing unjust or unfair wildlife management rules or regulations. ………

If not on the list, please indicate what you would prefer implemented.
……………………………………………………………………………………………………
……………………………………………………………………………………………………

Thank You Very Much
