# Supplementary material for: Persistent Illegal Hunting of Wildlife in an African Landscape: Insights from a Study in the Luangwa Valley, Zambia
Source: Animals (Basel). 2024 Aug 19;14(16):2401. doi: 10.3390/ani14162401 (PMC11350902; doi:10.3390/ani14162401)
Supplement: Supplementary file 1 [file animals-14-02401-s001.zip › Table S1 pdf.pdf]

Table S1. Thematic Data Analysis Results for Nine Focus Group Discussions (FGDs) and Three In-Depth Interviews (IDIs) Conducted in the Luangwa Valley. Figures Indicate Numbers of References for Themes or Subthemes and Figures in the Parentheses Show Numbers of FGDs and IDIs where Respective Themes were Discussed

Theme: Drivers of Illegal Hunting

	Protesting unfairness / injustices	Poverty	Inadequate law enforcement	Need for income	Human wildlife conflicts	Lack of alternative livelihood	Limited tangible benefits	Lack of employment	Inadequate community involvement	Demand/market for bushmeat or products	Lack of alternative meat/food	Limited access to bushmeat	Poor partnerships/ collaboration	Encroachment & development	Inadequate awareness / sensitisation	Human pop/ influx increase	Communities don't own wildlife
FGDs	19 (5)	14 (6)	11 (1)	10 (4)	10 (3)	8 (3)	7 (3)	6 (3)	4 (4)	1 (1)	1 (1)	1 (1)	1 (1)	0	0	0	0
IDIs	0	2 (1)	12 (3)	3 (2)	1 (1)	2 (1)	2 (2)	3 (1)	0	4 (3)	1 (1)	0	0	2 (2)	1 (1)	1 (1)	2 (1)

Theme: Intervention Measures

	Provision of alternative livelihoods	Improve benefits from wildlife	Provision of employment	Sensitisation awareness	Improving law enforcement	Involving Communities in conservation	Stakeholders collaboration	Human Wildlife Conflict mitigation	Provision of bushmeat or alternatives	Improving conservation funding
FGDs	21 (6)	12 (6)	9 (5)	5 (4)	5 (2)	4 (4)	4 (3)	2 (1)	2 (1)	0
IDIs	4 (1)	1 (1)	0	3 (3)	1 (1)	2 (2)	3 (2)	0	0	1 (1)

Theme: Unsatisfactory Intervention Performance

	Provision of alternative livelihoods	Provision of employment	Improving law enforcement	HW conflict mitigation	Bushmeat/ alternative meat provision	Provision/ access to tangible benefits	Conservation sensitisation/ awareness	Community involvement conservation
FGDs	14 (5)	12 (4)	12 (3)	7 (3)	5 (3)	4 (3)	1 (1)	1 (1)
IDIs	1 (1)	0	8 (3)	0	0	2 (2)	1 (1)	4 (2)

Theme: Behavioural Beliefs (attitude)

	Wildlife was given for use	Poaching is good for survival	Wildlife was given as food	Poaching is bad	God's gift for livelihood & survival	Wildlife is God's creation	Poaching, the only means for survival	Hunting is traditional	Wildlife brings wealth
FGDs	9 (4)	5 (4)	4 (3)	3 (3)	3 (2)	2 (2)	2 (1)	1 (1)	1 (1)

Theme: Normative Beliefs

	Poaching helps people to survive	Some may not support poaching	People support poaching
FGDs	5 (3)	4 (2)	4 (1)

Theme: Control Beliefs

	Easy to start poaching	Difficult to stop poaching	Easy to stop poaching
FGDs	12 (5)	6 (3)	3 (2)

Theme: Types of Illegal Hunters

	Local illegal hunters	Non-local
FGDs	20 (7)	2 (2)
IDIs	2 (2)	2 (2)

Theme: Limitations of Law Enforcement

FGDs	18 (6)
IDIs	6 (3)

Theme: Behavioural Intentions to Hunt Illegally

FGDs	17 (6)
