# Supplementary material for: Persistent Illegal Hunting of Wildlife in an African Landscape: Insights from a Study in the Luangwa Valley, Zambia
Source: Animals (Basel). 2024 Aug 19;14(16):2401. doi: 10.3390/ani14162401 (PMC11350902; doi:10.3390/ani14162401)
Supplement: Supplementary file 1 [file animals-14-02401-s001.zip › Table S2.pdf]

Table S2: Drivers of Illegal Hunting as Identified from Nine Focus Group Discussions (FGDs) and Three In-Depth Interviews (IDIs) Held in the Luangwa Valley.

Drivers of illegal hunting	No. of coding references (No. of FGDs or IDIs in parentheses)	Proximate/ underlying drivers	Thematic drivers
Weak/inadequate law enforcement	23 (FGD, 1; IDIs, 3)	Underlying	Inadequate legislation/ enforcement
Defiance/Protesting	19 (FGDs, 5)	Proximate	Defiance/protesting unfairness
Intention to hunt illegally	17 (FGDs, 6)	Proximate	Behavioural intention to hunt illegally
Poverty	16 (FGDs, 6; IDI, 1)	Underlying	Need for survival & sustaining livelihoods
Need for income from bushmeat & animal products	13 (FGDs, 4; IDIs, 2)	Proximate	Need for survival & sustaining livelihoods
Human-wildlife conflicts	11 (FGDs, 2; IDI, 1)	Underlying	Human-wildlife conflicts
Lack of alternative livelihoods	10 (FGDs, 3; IDI, 1)	Proximate	Need for survival & sustaining livelihoods
Lack/inadequate tangible benefits from conservation	9 (FGDs, 3; IDIs, 2)	Underlying	Need for survival & sustaining livelihoods
Inadequate employment opportunities	9 (FGDs, 3; IDI, 1)	Proximate	Need for survival & sustaining livelihoods
Market/demand for wildlife products	5 (FGD, 1; IDIs, 3)	Underlying	Market demand for wildlife products
Inadequate community involvement in wildlife management	4 (FGDs, 4)	Underlying	Inadequate devolution of wildlife management
Retaliatory killing	2 (FGDs, 2)	Proximate	Human-wildlife conflicts
Human encroachment / development	2 (IDIs, 2)	Underlying	Encroachment / development
Lack of sources of meat/protein	2 (FGD, 1; IDI, 1)	Proximate	Need for survival & sustaining livelihoods
Non wildlife ownership by communities	2 (IDI, 1)	Underlying	Non wildlife ownership by communities
Inadequate access to legal bushmeat	1 (FGD, 1)	Proximate	Need for survival & sustaining livelihoods
Human population influx / increase	1 (IDI, 1)	Underlying	Demographic growth
Poor collaboration/ partnership	1 (FGD, 1)	Underlying	Inadequate collaboration
